# Tobacco and Substance Use among Psychiatric Inpatients in a Community Hospital: Cessation Counseling, Correlates, and Patterns of Use

**DOI:** 10.1155/2018/7919704

**Published:** 2018-12-18

**Authors:** Oluwole Jegede, Olawale Ojo, Saad Ahmed, Kodjovi Kodjo, Inderpreet Virk, Dina Rimawi, Cory Mellon, Ayodeji Jolayemi, Tolu Olupona

**Affiliations:** ^1^Interfaith Medical Center, Brooklyn, New York, USA; ^2^American University of Antigua, Antigua and Barbuda

## Abstract

**Background:**

Epidemiological and experimental models have been applied to describe the disproportionately high prevalence of tobacco use in patients with mental illness. This observed association has become a dire public health concern. The main objective of the present study was to examine the provision of tobacco treatment strategies in a community teaching hospital serving a predominantly underserved African American population.

**Methods:**

The study was designed as a retrospective review of eight hundred and thirty patients admitted to the inpatient psychiatric units.

**Results:**

52.2% of the entire cohort described themselves as current smokers. Gender, primary psychiatric diagnosis, and urine toxicology showed significant differences in the tobacco smoking and nontobacco smoking groups (P<0.05). Almost all current tobacco smokers (91.9%) had tobacco cessation counseling during the course of their hospitalization, but only 64% were offered treatments for tobacco dependence. More than half (57.9%) of the 680 participants who had urine toxicology reports were positive for any illicit substance with cannabis and cocaine being the most frequently used (32.4% and 23.2%). Direct logistic regression revealed gender, psychiatric diagnosis, and substance use as the only significant predictors of tobacco smoking among our cohort (P= 0.021, 0.001, and 0.001, respectively).

**Conclusions:**

Tobacco screening, cessation counseling, and treatment continue to be a challenge in community psychiatric hospitals and need increased focus in the comprehensive management of patients with psychiatric disorders. The strong association between tobacco smoking and other substance use lends itself to the hypothesis that tobacco smoking debut prevention may be an effective public health strategy for addressing illicit drug use.

## 1. Introduction

Tobacco use among individuals with mental illness continues to be a subject of increasing scientific inquiry over the last several decades as epidemiologic and experimental literature have attempted to describe and unravel the disproportionate impact of tobacco use disorder in this demographic. Among individuals with mental illness, tobacco smoking presents peculiar challenges, one of which is due to its direct influence on the pharmacokinetics (and efficacy) of many psychotropic medications, thus negatively impacting patients' quality of life. In addition, tobacco smoking is a known modifiable and preventable risk factor for cardiometabolic morbidity and mortality [1, 2].

The lifetime prevalence of substance use among patients with schizophrenia is conservatively reported at 47% [3]. Although significant declines have been reported in the smoking prevalence among the general population, this is not the case in patients with the mentally ill population, where the rates have remained unchanged and on an upward trajectory [1]. In 2016, the prevalence of current cigarette smoking among adults was 15.5%, a decline from 2005 (20.9%), but since 2015 no significant changes have occurred with the smoking prevalence reported to be around 15.1%. The Centers for Disease Control and Prevention (CDC) report noted quite succinctly that, among people with “serious psychological distress”, the prevalence of smoking continues to increase significantly. They further stressed the need for population and sub-population-based interventions at reducing tobacco-related diseases among US adults [4, 5].

According to the Substance Abuse and Mental Health Services Administration (SAMHSA's) 2014 National Survey on Drug Use and Health (NSDUH), among the 20.2 million adults with a past year history of substance use, 7.9 million (39.1 percent) had any mental illness in the past year, but among adults without substance use disorder in the past year, only 16.2 percent had any mental illness in the past year, further suggesting a relationship between substance abuse and mental illness [6].

Several biopsychosocial hypotheses have been proposed to explain the particular vulnerability to substance use in patients with mental illness. The biological and natural course of mental illness suggests that patients may be self-medicating their symptoms or the adverse effects of their psychotropic medications as they seek to increase dopamine in the brain reward centers and prefrontal cortex [3]. The shared biological vulnerability between schizophrenia and substance abuse has been investigated to explain common genetic determinants between these conditions and their shared dopaminergic and opioid receptor etiologies [7]. Socially, like the general population, substance use may be the gateway for acceptance into certain social groups, but some authors have also argued that this “social acceptance theory” may, in fact, be a chance occurrence given that the age in which most mental illness manifests ais also the age range when most people are likely to begin using illicit drugs [2, 3].

Tobacco treatment (TT) has been shown to produce remarkable economic benefits and cost savings, reduce the rate of rehospitalizations, and improve health outcomes such as mental and physical functioning among mentally ill patients [8, 9]. Furthermore, the role of TT in improving the cognitive function of patients with mental illness was the highlight of a new study by Vermeulen et al., 2018, which linked tobacco smoking with poorer cognitive performance in patients with mental illness compared with nonsmoking controls and concluded that tobacco smoking cessation may improve processing speed and cognition [10]. Unfortunately, behavioral health settings across the United States continue to report the relatively low availability of evidence-based tobacco cessation treatments and smoke-free environments according to a recent CDC report [11]. In the same report, 49% of mental health facilities screened patients for tobacco use, 38% offered tobacco cessation counseling, and 49% had smoke-free campuses [11].

Several studies have shown, conclusively, that adolescent exposure to nicotine increases susceptibility to addiction to other substances such as alcohol, cocaine, methamphetamine, and opioids [11, 12]. There is also an increased recognition that this association (between tobacco use initiation and subsequent substance abuse) could be regarded as a primary substance abuse prevention strategy especially in the current age of the opioid epidemic [12]. In addition, the interaction of chronic pain and tobacco use is suggested as a possible mechanism for increased opioid abuse among this population [13].

There is overwhelming evidence of the efficacy of tobacco screening and cessation counseling in helping tobacco users quit. The United States Preventive Services Task Force (USPSTF) recommends that clinicians ask all adults about tobacco use, advise them to stop using tobacco, and provide behavioral interventions and pharmacotherapy [14]. This present study adds to existing data on the provision of tobacco cessation strategies including counseling, nicotine replacement treatments, and nonnicotine medications in a community teaching hospital serving a predominantly underserved African American community. The study also aims to determine predictive relationships between various variables such as patient-related demographic variables, psychiatric diagnosis, other illicit drug use, and tobacco consumption. Finally, we described some patterns of substance use among psychiatric inpatients in our facility.

## 2. Methods

This study is designed as a retrospective review of all the charts of patients admitted to the inpatient psychiatric units of a community teaching hospital. Eight hundred and thirty charts were reviewed on a case by case basis (patients were admitted between July and November 2017). Patients' personal information was deidentified and the following information was extracted from the charts: age, gender, race, the primary psychiatric diagnosis, urine toxicology report (indicating substance use), the type of cessation counseling provided, and, finally, the type of intervention provided to the patients such as Nicotine Replacement Therapy (NRT) and nonnicotine medications. The study was approved by the Institutional Review Board (IRB) of Interfaith Medical Center, Brooklyn, New York, United States.

### 2.1. Statistical Analysis

All variables (continuous, categorical, and dichotomous) were outlined in a codebook with dummy variables created for statistical analysis. Data were analyzed using the IBM SPSS Statistics version 25. Descriptive statistics of means and standard deviations were used to describe all continuous variables and the frequencies of all the other categorical variables were calculated and reported. Chi-square statistics was used to determine any significant associations between selected categorical variables and smoking status. Logistic regression was performed to assess the impact of selected independent variables: age, gender, race, primary psychiatric diagnoses, and urine toxicology on smoking status. The statistical significance was set at P<0.05.

## 3. Results

### 3.1. Baseline Characteristics

Eight hundred and thirty subjects, aged between 18 and 84 years old, the mean age 42.14 ± 13.3 years, participated in this study. Other demographic information is as shown in Table 1; of note, in our cohort, the majority of our patients were males and African Americans, 66.1% and 72.7%. Our results show that only 52.2% described themselves as current smokers. Gender, psychiatric diagnosis, and urine toxicology differed significantly in relation to smoking status (P<0.05).

### 3.2. Tobacco Treatment

**Figure 1:**
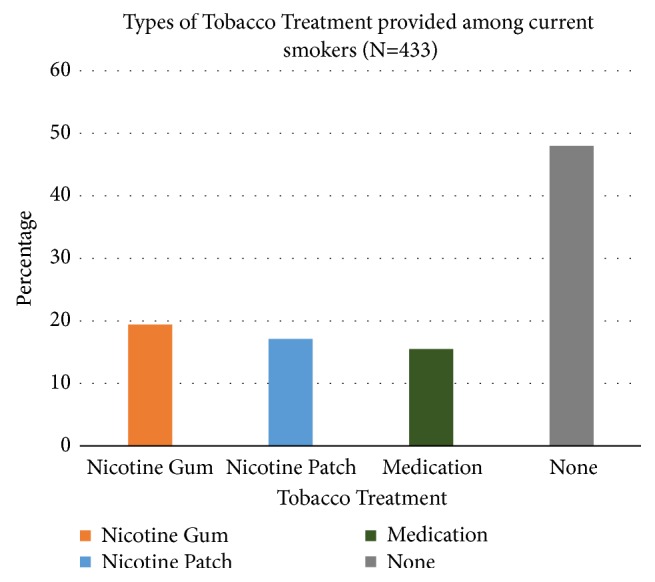
The type of tobacco treatment provided.

91.9% of current smokers (N=433) compared to 48.3% of the entire population studied (N=830) had a documented tobacco cessation counseling done at any period during their hospitalization. 64% of current smokers were offered any treatment for tobacco dependence at any time during their hospitalization in the form of nicotine gum, nicotine patches, or medications including Bupropion SR and Varenicline. The distribution of the forms of tobacco treatment is provided in Figure 1.

### 3.3. Urine Toxicology

**Figure 2:**
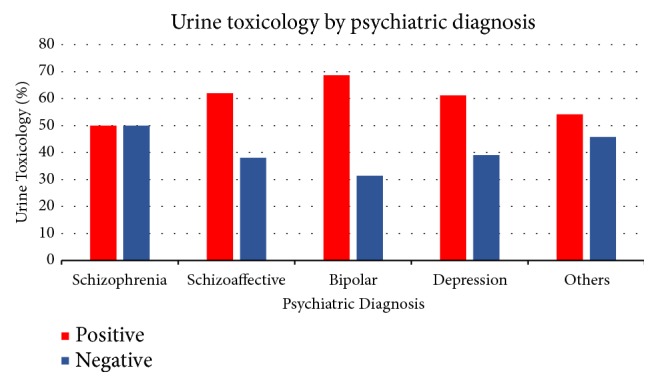
The distribution of urine toxicology report by psychiatric diagnoses.

The urine toxicology reports of the cohort were recorded as positive, negative, or “not reported”. 57.9% of reported urine toxicology reports were positive for any substance; of these, cannabis and cocaine were the most frequently used drugs (32.4% and 23.2%). The frequency of urine toxicology positive for opioid, PCP, and alcohol was 2.9, 1.2, and 3.7%, respectively.  Table 2 shows the distribution of substance use by the primary psychiatric diagnoses and Figure 2 shows the urine toxicology as distributed by patient diagnoses. Of all the substances, only cocaine and alcohol showed significant association with the patient psychiatric diagnosis P = 0.001 and P = 0.023, respectively (Table 2). It is interesting to note that only 12.5% of the population had urine toxicology positive for more than one substance.

### 3.4. Logistic Regression and Chi-Square Analysis

Our statistical model was determined using direct logistic regression analysis, the dependent variable being the dichotomous tobacco smoking status and independent predictors being age, gender, race, primary psychiatric diagnosis, and urine toxicology. The model with all five predictors was statistically significant *χ*^2^ (11, N=830) = 75.580, p<0.05, showing that this model was able to distinguish between smokers and nonsmokers in our sample. Our model explained between 8.5% (Cox and Snell R square) and 11.3% (Nagelkerke R square) of the variance in the smoke status and correctly classified 64.3% of cases. As shown in Table 3 gender, psychiatric diagnosis, and substance use (urine toxicology) were significant predictors of tobacco smoking. Urine toxicology, however, provided the strongest contribution (OR = 2.70) indicating over twice likelihood of individuals with a positive urine toxicology to report smoking cigarette, controlling for other factors in the model.

A stepwise Chi-Square analysis for independence (with Yates continuity correction) indicated a significant association between each of the variables: gender, psychiatric diagnosis, and urine toxicology with tobacco smoking but failed to report any association with age, race, or any other variables in the model. Pearson Chi-Square values and symmetric measures, Phi coefficient, and Cramer's V showing effect sizes are reported in Table 4.

## 4. Discussion

The results of our study should be interpreted in the context of the study design and study setting. Our institution is a community teaching hospital located in the Bedford Stuyvesant community of Brooklyn, New York, serving a predominantly African American population. The hospital has a 90-bed inpatient unit, an inpatient detoxification unit, and drug rehabilitation units, and a psychiatric emergency service. The population studied was predominantly males (66.1%) and African Americans (72.7%). The mean age of the 830 individuals was 42.14 ± 13.3 years. The overarching goal of the present study was to assess tobacco screening strategies including cessation counseling and interventions among psychiatric inpatients in our hospital and to assess the influence of substance abuse and patient-related factors on their tobacco smoking status. Our results compare quite favorably with other similar studies; it is interesting to note that most of the few available community-based studies were conducted in predominantly Caucasian populations. The age of our population was similar to that of Collins et al. (2018) and Okoli et al. (2018) (mean age 41.2 years); the Collins study reported 49.8% tobacco smokers in the emergency room setting and Okoli reported 59% (compared to 52.2% in our study). Although both studied predominantly Caucasian populations, the similarities of some of the results with ours are rather striking [14, 15].

Carrillo, 2017, in a retrospective chart review of inpatient psychiatric admission reported an 18-fold increase in referral to cessation counseling (from 4% to 74%) and referral to cessation medication (from 32% to 68%) after the implementation of the Centers for Medicare & Medicaid Services (CMS) policy that required reporting of tobacco treatment quality measures [16]. In our study, almost all the smokers, 91.9%, (N=433) had tobacco cessation counseling done, but only 64% of that population was offered any tobacco treatment.

Another significant result of our study is the strong association between tobacco smoking and each of gender, psychiatric diagnosis, and substance use (p= 0.05, 0.001, 0.001, respectively). The association between tobacco smoking and other substance use is of particular significance especially as the opioid epidemic has become a national catastrophe and a public health crisis. In discussing this association, Marynak and colleagues, 2018, opined that the phenomenon “reinforces the importance of tobacco prevention and cessation efforts across the lifespan” [10]. They further stated that tobacco initiation prevention may be viewed as a way to curb subsequent substance abuse. In our study, 57.9% of our patients had a positive urine toxicology; of these, cannabis and cocaine were the most frequently used drugs at 32.4% and 23.2%, which is not surprising given our population of 58% with a diagnosis of schizophrenia and schizoaffective disorders. We can hypothesize that in patients with psychiatric disorders, perhaps tobacco smoking may be a gateway to other illicit drugs; further studies will be needed to explore this hypothesis.

A rather unexpected finding in this study is the difference in the frequency of substance use and tobacco smoking among individuals with a diagnosis of schizoaffective disorder and schizophrenia. Among those with schizoaffective disorder, the numbers are 62% and 33.7% (for substance use and tobacco smoking) compared to those with schizophrenia at 50% and 25.6%, respectively, thus showing a consistently higher frequency among schizoaffective than schizophrenia. Possible explanations for this difference, although not statistically significant (P>0.05), may be the artifact of diagnostic practices and, perhaps, the significant role of the mood component in schizoaffective disorder.

Our study is not without limitations, like other studies that utilize the retrospective design; we were limited by our inability to control variables; possible incomplete documentation in the electronic medical records and underreporting of substance and tobacco use are inherent. The interpretation and generalizability of our results should also be done in the context of the population we serve. Although being a retrospective study, we found our choice of urine toxicology, as the index of substance use, to be very useful; a prospective study that assesses other areas of substance use disorder may provide more information about dependence and other patterns of drug use. Finally, we were unable to account for other substances (such as synthetic cannabinoids) that are not routinely tested on urine toxicology but may nonetheless provide interesting insights into studies of this nature.

## 5. Conclusions and Future Directions

Tobacco screening, cessation counseling, and treatment continue to be a challenge in community psychiatric hospitals. Models of comprehensive management of patients with psychiatric disorders must thus, invariably include a renewed focus on issues of tobacco screening, counseling and treatments. In addition, the strong association between tobacco smoking and substance use lends itself to the hypothesis that strategies to prevent tobacco smoking debut may be considered as possible primary prevention of subsequent development of other substance use disorders in these patients. Further studies with a prospective design that control for more variables, assess patterns of drug use through direct interviews, and include continuous variables such as number of cigarettes smoked and package years, may address more questions than we could answer given the limitations of this study. Finally, longitudinal studies to further test the temporal relationship between tobacco and other substance use (including synthetic cannabinoids and other designer drugs) may show new and innovative ways to tackle the global epidemic of drug abuse.

## Figures and Tables

**Table 1:** Tobacco smoking status distribution according to demographic characteristics, primary psychiatric diagnosis, and urine toxicology.

**Patient Characteristics**	**Tobacco Smokers** **(N/**%**)**	**Nontobacco Smokers (N/**%**)**
Sex*∗*		
Male	306 (70.7)	243 (61.2)
Female	127 (29.3)	154 (38.8)

Race		
African American	322 (74.4)	281 (70)
Asian	10 (2.3)	12 (3.0)
Hispanic	55 (12.7)	57 (14.3)
White	17 (3.9)	19 (4.8)
Other	29 (6.7)	28 (7.0)

Psychiatric Diagnosis*∗*		
Schizophrenia	111 (25.6)	138 (34.8)
Schizoaffective	146 (33.7)	86 (21.7)
Bipolar	69 (15.9)	54 (13.6)
Depression	36 (8.3)	41 (10.3)
Others	71 (16.4)	78 (19.6)

Urine Toxicology*∗*		
Positive	256 (30.8)	138 (16.6)
Negative	113 (13.6)	173 (20.8)
No data available	64 (7.7)	86 (10.4)

Total	433 (52.2)	397 (47.8)

*∗*P<0.05.

**Table 2:** The distribution of substances by psychiatric diagnosis (N= 431).

**Urine Toxicology (Substance Use)**	**Psychiatric Diagnosis**	**N**
Cannabis	Schizophrenia	57
	Schizoaffective	60
	Bipolar	39
	Depression	20
	Others	44

Opioid	Schizophrenia	1
	Schizoaffective	5
	Bipolar	6
	Depression	4
	Others	4

Cocaine*∗*	Schizophrenia	27
	Schizoaffective	60
	Bipolar	27
	Depression	21
	Others	23

PCP	Schizophrenia	1
	Schizoaffective	2
	Bipolar	1
	Depression	0
	Others	4

Alcohol*∗*	Schizophrenia	2
	Schizoaffective	9
	Bipolar	5
	Depression	7
	Others	2

*∗*P< 0.05.

**Table 3:** Logistic regression analysis of variables.

	B	S.E.	Wald	df	Sig.	Exp(B)	95% C.I. for EXP(B)	
							Lower	Upper
Age	.000	.006	.002	1	0.966	1.000	.989	1.011
*∗*Gender	.360	.157	5.295	1	**0.021**	**1.434**	**1.055**	**1.949**
Race	-.206	.453	.207	1	0.649	.814	.335	1.977
*∗*Psychiatric diagnosis	.738	.196	14.239	1	**0.001**	**2.091**	**1.426**	**3.068**
*∗*Urine Toxicology	.994	.166	35.837	1	**0.001**	**2.701**	**1.951**	**3.739**
Constant	-.898	.303	8.783	1	0.003	0.408		

**Table 4:** Chi-Square analysis of selected variables and smoking status.

Variables	X^2^	P	Phi^a^/Cramer's V^b^
Gender *∗* Smoking status	7.861	**0.05**	0.1^a^
Race *∗* Smoking status	1.575	0.813	0.044^b^
Diagnosis *∗* Smoking status	19.403	**0.001**	0.153^b^
Urine Toxicology *∗* Smoking status	46.96	**0.001**	0.245^b^

## Data Availability

The data used to support the findings of this study are available from the corresponding author upon request.

## References

[1] Jamal A., Phillips E., Gentzke A. S. (2018). Current Cigarette Smoking Among Adults — United States, 2016. *Morbidity and Mortality Weekly Report (MMWR)*.

[2] Akerele E. O., Levin F. R. (2002). Substance Abuse Among Patients with Schizophrenia. *Journal of Psychiatric Practice*.

[3] Buckley P. F., Miller B. J., Lehrer D. S., Castle D. J. (2009). Psychiatric comorbidities and schizophrenia. *Schizophrenia Bulletin*.

[4] CDC. Best practices for comprehensive tobacco control programs—2014. Atlanta: US Department of Health and Human Services, CDC, National Center for Chronic Disease Prevention and Health Promotion, Office on Smoking and Health, 2014. https://www.cdc.gov/tobacco/stateandcommunity/best_practices/index.htm

[5] US Department of Health and Human Services. The health consequences of smoking—50 years of progress: a report of the Surgeon General. Atlanta, GA: US Department of Health and Human Services, CDC, Coordinating Center for Health Promotion, National Center for Chronic Disease Prevention and Health Promotion, Office on Smoking and Health, 2014. http://www.surgeongeneral.gov/library/reports/50-years-of-progress/full-report.pdf

[6] Center for Behavioral Health Statistics and Quality (2015). *Behavioral health trends in the United States: Results from the 2014 National Survey on Drug Use and Health (HHS Publication No. SMA 15-4927, NSDUH Series H-50)*.

[7] Batel P. (2000). Addiction and schizophrenia. *European Psychiatry*.

[8] Taylor G., McNeill A., Girling A., Farley A., Lindson-Hawley N., Aveyard P. (2014). Change in mental health after smoking cessation: systematic review and meta-analysis. *BMJ*.

[9] McCallum D. M., Fosson G. H., Pisu M. (2014). Making the case for Medicaid funding of smoking cessation treatment programs: An application to State-Level health care savings. *Journal of Health Care for the Poor and Underserved*.

[10] Vermeulen J. M., Schirmbeck F., Blankers M. (2018). Association Between Smoking Behavior and Cognitive Functioning in Patients With Psychosis, Siblings, and Healthy Control Subjects: Results From a Prospective 6-Year Follow-Up Study. *The American Journal of Psychiatry*.

[11] Marynak K., VanFrank B., Tetlow S. (2018). Tobacco Cessation Interventions and Smoke-Free Policies in Mental Health and Substance Abuse Treatment Facilities — United States, 2016. *Morbidity and Mortality Weekly Report (MMWR)*.

[12] US Department of Health and Human Services (2016). Health effects of ecigarette use among U.S. youth and young adults (chapter 3). *E-cigarette use among youth and young adults: a report of the Surgeon General*.

[13] Yoon J. H., Lane S. D., Weaver M. F. (2015). Opioid Analgesics and Nicotine: More Than Blowing Smoke. *Journal of Pain and Palliative Care Pharmacotherapy*.

[14] Siu A. L. (2015). Behavioral and pharmacotherapy interventions for tobacco smoking cessation in adults, including pregnant women: U.S. preventive services task force recommendation statement. *Annals of Internal Medicine*.

[15] Collins A., Ajayi O., Diamond S., Diamond W., Holroyd S. (2018). Tobacco Use and Associated Factors in Patients Presenting to a Psychiatric Emergency Room. *Journal of Addiction Medicine*.

[16] Carrillo S., Nazir N., Howser E. (2017). Impact of the 2015 CMS inpatient psychiatric facility quality reporting rule on tobacco treatment. *Nicotine & Tobacco Research*.

